# Current Endoscopic and Surgical Management of Pancreatic Cystic Lesions: A Comprehensive Review

**DOI:** 10.5152/tjg.2024.24124

**Published:** 2025-01-01

**Authors:** Kunzah A. Syed, Kelly J. Lafaro, Elham Afghani

**Affiliations:** 1 Division of Gastroenterology, Department of Medicine, Johns Hopkins Medical Institutions, Baltimore, Maryland, USA; 2 Department of Medicine, Pancreatitis Center, Johns Hopkins Medical Institutions, Baltimore, Maryland, USA; 3 Division of Hepato-Pancreato-Biliary Surgery, Department of Surgery, Johns Hopkins University School of Medicine, Baltimore, Maryland, USA

**Keywords:** Pancreatic cyst,, endosonography,, surgical procedures,, gastrointestinal endoscopy,, pancreatic neoplasms

## Abstract

Pancreatic cystic lesions (PCLs) are frequent incidental findings with an increasing prevalence with age. For these significant lesions, accurate characterization of cyst type and prediction of the risk of malignant progression are crucial for specific management, such as deciding to monitor a lesion or pursue surgical intervention. Fortunately, endoscopy-based diagnostic and therapeutic techniques like endoscopic ultrasound with fine needle aspiration, confocal endomicroscopy, through-the-needle biopsy, contrast-enhanced endoscopic ultrasound, ablation, and pancreatoscopy have enabled increasingly accurate diagnoses of PCLs. Surgical management should be considered in certain cases. This narrative review’s objective is to appraise and synthesize the salient literature on the endoscopic and surgical management of PCLs to aid clinician decision-making. We analyze the current data and explore the benefits, challenges, and future prospects of endoscopy and surgery for pancreatic cysts.

**Main Points**

- Pancreatic cystic lesions (PCLs) are common, increase in prevalence with age, and have a variable risk of cancer progression. Determining the type of cyst is imperative for appropriate management.
- The proper management of PCLs involves weighing numerous guidelines, with the caveat that they are based on a low level of evidence.
- Since imaging techniques are imperfect, endoscopic techniques aim to provide an improved method of differentiating PCLs.
- There are many nuances to the surgical resection of PCLs and various indications, which range from relative to absolute. Surgical indications should be carefully considered as they entail significant morbidity and mortality.

## Introduction

Pancreatic cystic lesions (PCLs) are abundant and typically incidentally detected. Prospective data show that the prevalence of PCLs ranges up to 49%^1^ and increases with age.^2^ The incidence varies depending on the imaging modality, with magnetic resonance imaging (MRI) detecting more cystic lesions when compared to computed tomography (CT) scan.^2^ The natural history of PCLs depends on the nature of the cyst, as they can be benign, premalignant, or malignant. Distinct imaging features can suggest a diagnosis of the type of cyst and its risk of malignant progression; however, an abundance of overlapping features challenges accurate characterization. Determining the type of cyst is key to appropriate management. Information about cyst morphologies is represented in Table 1.

Cysts can be categorized based on the likelihood of malignant transformation: no malignant potential, malignant potential, and malignant. Lesions which lack significant malignant potential are pseudocysts, serous cystadenomas (SCAs), lymphoepithelial cysts, congenital cysts, retention cysts, and lymphangiomas. Cysts of this category can safely be left alone without surveillance. Cysts with malignant potential, i.e., intraductal papillary mucinous neoplasms (IPMNs) and mucinous cystic neoplasms (MCNs), have a varying risk of malignant progression, and their risk necessitates whether monitoring or surgical resection should be considered. There are three types of IPMNs: branch-duct IPMNs (BD-IPMNs), which have a lower risk of malignant transformation, main duct IPMNs (MD-IPMNs), which have a higher risk, and mixed-type IPMNs, which are thought to have a higher risk compared to BD-IPMNs.^3^ Clinicians rely on certain symptoms and imaging features to predict the risk of malignant transformation. High-risk stigmata (HRS) include obstructive jaundice, dilated main pancreatic duct ≥10 mm and/or enhancing mural nodule ≥5 mm, and association with the highest risk of malignancy. Worrisome features (WF) of mucinous cysts are a main pancreatic duct (MPD) size of 5-9 mm, thickened or enhancing cyst walls, presence of a mural nodule <5 mm in size, cyst size >3 cm (for IPMNs), cyst growth surpassing 3 mm per year or >5 mm in 2 years, abrupt change in caliber of the pancreatic duct with distal pancreatic atrophy, or positive cytology.^3^ Notably, the most recent International Association of Pancreatology Guidelines now include new-onset or acute exacerbation of diabetes mellitus (DM) in the past 12 months in WF.^2^ A recent large, multicenter study of 810 patients with IPMNs who underwent pancreatic resection found that 63% of those with one HRS had malignant progression on pathology after resection compared to 100% in those with more than 2 features. Twenty-two percent of individuals with one WF were found to have malignant progression. The risk of malignancy increased in a stepwise fashion with the number of WF present, reaching 100% in those with more than 3 WF.^4^ Mucinous cystic neoplasms that are <4 cm are considered to have lower rates of malignant progression, with one systematic review reporting the risk as 0.03%.^5^ Cystic lesions that are malignant from the outset, such as solid pseudopapillary neoplasms (SPN), cystic degeneration of pancreatic adenocarcinoma, or certain pancreatic neuroendocrine tumors (PanNET), are recommended to be addressed with surgery. The diagnosis of PCLs is challenging because there is a nearly 30% discrepancy between pre- and post-diagnosis in those who have undergone pancreatic resections.^6^ This is further limited by the low diagnostic accuracy of conventional cross-sectional imaging. A meta-analysis of 28 studies comparing modalities found that the pooled sensitivities for CT, MRI/magnetic resonance cholangiopancreatography, and endoscopic ultrasound (EUS) in PCL differentiation were 0.7, 0.76, and 0.6, respectively, while the pooled specificities were 0.78, 0.83, and 0.8.^7^ Therefore, imaging alone is often insufficient for high diagnostic certainty. Endoscopic ultrasound is a necessary adjunct and logical next step in evaluation.

## Endoscopic Ultrasound

Compared to conventional cross-sectional imaging, EUS is superior and provides high-quality images in the diagnosis and evaluation of PCLs, especially when the diagnosis is uncertain or WF are present.^8^ The diagnostic accuracy of diagnostic EUS for identifying mucinous cysts has been demonstrated to be as low as 40% but as high as 94%.^9^ Nevertheless, EUS can provide a better assessment of mural nodules and thickened septa than cross-sectional imaging. Endoscopic ultrasound as a modality allows proceduralists to distinguish a mural nodule, which is a concerning feature, from a mucin globule or ball, which is a benign feature. This is discerned based on echogenicity, with a mural nodule being iso- or hyperechoic and accompanied by irregular margins.^10^ A mucin globule contains a hypoechoic center enclosed by a well-demarcated, hyperechoic rim. A mucin ball can be recognized by its likelihood of shifting in response to a patient’s position changes.^8^ This feature is often also utilized in the evaluation of main duct dilation that may be suggestive of main duct or mixed-type IPMN, in order to rule out an obstructing lesion causing secondary ductal dilation. Endoscopic ultrasound provides excellent contrast and spatial resolution imaging but is operator-dependent with fair interobserver agreement at best, as denoted by a maximum kappa value of 0.53.^10^ Operator-dependency and modest interobserver agreement are considered downsides. Additionally, it is an invasive and costly option. Regardless, EUS has benefits and, while it cannot be used for every patient, it is recommended in the European,^11^ American College of Gastroenterology,^12^ and nternational Association of Pancreatology (IAP) guidelines^2^ if it will change management and/or there is suspicion of high-grade dysplasia (HGD) or invasive carcinoma.

### Endoscopic Ultrasound with Fine Needle Aspiration

One of the most valuable assets of EUS is its ability to attain fluid for cytologic and molecular marker assessment. Endoscopic ultrasound with fine-needle aspiration (EUS-FNA) is integral to endoscopic management for the accurate characterization of cyst type by assessing biomarkers within the cyst fluid, as well as determining HGD or malignancy.^8^ A study evaluating the performance characteristics of EUS with or without FNA found that EUS-FNA was superior to CT and MRI in the accurate classification of cysts as neoplastic and also incrementally increased the rate of correct prediction of neoplastic cysts.^13^ Workup with EUS-FNA should be preferentially undertaken at centers where experts are present.^3,14^ Overall, FNA is low-risk and associated with a morbidity of up to 2.5%. Its main complications are acute pancreatitis, bleeding, and infection,^15^ as well as those associated with the anesthesia administered. In a systematic review, the rates of pancreatitis, bleeding, and infection did not exceed 0.44% and the demonstrated mortality was 0.02%.^16^

### Cyst Fluid Analysis

Obtaining cyst fluid for analysis provides valuable adjunctive diagnostic information through interpretation of cytology, carcinoembryonic antigen (CEA) and amylase levels, glucose concentration, and molecular markers. Besides during EUS-FNA, cyst fluid can be obtained during pancreatoscopy or intraoperatively. Cytology obtained from the cyst fluid helps separate benign cysts from precancerous or cancerous lesions. Cytologic diagnosis enables a preoperative diagnosis and can dictate surgical management. Pancreatic amylase is an enzyme secreted by the pancreas and released from the pancreatic ductal system into the small intestine. An amylase of <250 U/L helps exclude inflammatory pseudocysts with a specificity of 98%.^17^ However, amylase should not be used to discern MCNs from IPMNs.

In contrast, a higher CEA level is indicative of a mucinous cyst. Setting a threshold of >192 ng/mL results in a pooled specificity and sensitivity of 87% and 58%, respectively, based on a recent systematic review and meta-analysis. A low intracystic glucose level of <50 mg/dL is also sensitive for distinguishing benign, non-mucinous cysts from mucinous PCLs conferring malignant potential.^18^ Novel cut-offs have been proposed through co-analysis of cyst fluid CEA with a cut-off of 135.1 ng/mL and a glucose cut-off of 2.8 mmol/L to rule in mucinous neoplastic PCLs. To rule out mucinous PCLs, co-analysis of CEA using a cut-off of 6.12 ng/mL and a glucose cut-off of 2.8 mmol/L added value to prediction with a specificity of 93.3%.^19^

Finally, molecular marker analysis is a burgeoning field of increasing importance. Genetic profiles play a role in the recognition of cyst types. Molecular analysis can aid in identifying SCAs over IPMNs or MCNs, and in malignant progression. The presence of a *KRAS* and/or *GNAS* mutation has been associated with a 79% sensitivity and 98% specificity in diagnosing a mucinous cyst, whereas a von Hippel–Lindau (*VHL*) mutation has over 99% specificity in identifying an SCA. Mutations in *CDKN2A*, *PIK3CA*, *SMAD4*, and *TP53* have specificities of 97%, 97%, 98%, and 95%, respectively, in identifying malignant progression but at the cost of low sensitivities.^18^

Though molecular markers have proven useful in determining malignant progression, cystic CEA and glucose have no role. Useful cut-offs for the interpretation of cyst fluid analysis are summarized in Table 2. Limitations of molecular marker use include sampling error and low negative predictive value, depending on the biomarker used for assessment. Further limitations of molecular marker analysis include lack of access.

## Needle-Based Confocal Laser Endomicroscopy

Needle-based confocal laser endomicroscopy (nCLE) uses real-time visualization of PCLs. This advanced imaging technique entails directing a laser toward tissue taken from the gastrointestinal mucosa to facilitate the acquisition of high-quality, high-resolution images of cysts by passing a miniature confocal probe through a standard 19-gauge FNA needle.^20^ An in-vivo imaging tool, nCLE used adjunctively with EUS-FNA can be especially useful after imaging and cyst fluid analysis have not cinched a diagnosis. It can detect the superficial vascular network or fern-pattern specific for SCAs, fingerlike papilla in IPMNs, the presence of mucin epithelial bands characteristic of MCNs, dark neoplastic cell clusters with white fibrous bands in cystic NETs, and bright particles in pseudocysts.^21,22^ The presence of a superficial vascular network has 100% specificity and positive predictive value for diagnosing SCAs.^21^ When epithelial villous structures are visualized, the specificity for mucinous cysts or adenocarcinoma is 100%.^8^ It has a diagnostic accuracy of 99%. Moreover, the interobserver variability is substantial, with a kappa of 0.77.^21^ However, more evidence is likely needed to strengthen the interobserver variability. While generally safe, complications include a post-procedure pancreatitis prevalence of 1%, according to a 2022 systematic review and meta-analysis.^20^ The current guidelines for the management of pancreatic cysts do not recommend nCLE, although it is promising.

## EUS-Guided Through-the-Needle Biopsy

EUS-guided through-the-needle biopsy (EUS-guided TTNB) is another diagnostic innovation that can improve the differential diagnosis of PCLs. To avoid the need for unnecessary indefinite surveillance in the context of indeterminate findings, EUS-guided TTNB can be used to obtain histologic information that cross-sectional imaging, EUS morphology alone, and/or cystic fluid analysis cannot provide. A cyst’s wall, septa, or mural nodule can directly be sampled with microforceps to obtain a larger specimen. The ultimate goal of EUS-guided TTNB is to improve diagnostic accuracy via histologic diagnosis. A systematic review found that EUS-guided TTNB has a technical success rate of 98.5%, an overall diagnostic yield of 68.6%, and an adverse event rate of 9.7%.^23^ Adverse effects such as pancreatitis and intracystic bleeding were reported in up to 22% of patients.^24^ Additionally, a large retrospective multicenter analysis of TTNB patients described age, number of needle passes, complete cyst aspiration, and diagnosis of IPMN as independent predictors of adverse events.^25^ The adverse event rate comprises a safety concern. Currently, there is insufficient data on EUS-guided TTNB to delineate its role in the treatment algorithm of PCLs; however, recent evidence highlights its potential as a minimally invasive technique with an acceptable safety profile and high accuracy.^26^ However, the technique is considered investigational at best due to limited and mostly low-quality evidence.

## Contrast-Enhanced Harmonic EUS

Contrast-enhanced EUS (CE-EUS) is a non-invasive modality that uses microbubble contrast media to better inspect features of abdominopelvic anatomy. No significant difference was demonstrated between contrast-enhanced harmonic EUS, CT, and MRI in their ability to detect size and ductal dilatation.^27^ Importantly, the latest evidence-based guidelines from the IAP incorporate CE-EUS images as an essential method of assessing WF and HRS.^2^ The use of CE-EUS allows better visualization of small vessels and parenchymal enhancement. This can help differentiate the adherent necrotic debris seen in pseudocysts from an enhancing mural nodule. It can also aid in distinguishing a mucin ball from a mural nodule by improving the diagnostic accuracy from 50%-55% to 94%.^9,28^ A systemic review and meta-analysis evaluated the diagnostic performance of CE-EUS. The technique improved the diagnostic yield of finding and characterizing malignant mural nodules.^29^ With contrast-harmonic mode enabled during EUS, the sensitivity and negative predictive value are both 100%, while the accuracy is 94%.^28^ Given its high resolution, CE-EUS increases the sensitivity of EUS-FNA and may be used more widely in the near future.^30^ However, a recent network meta-analysis of 3641 patients determined that, at expert centers with relevant facilities, EUS-TTNB and EUS-nCLE were better options compared to CE-EUS for PCL diagnosis.^31^

## Pancreatoscopy

Pancreatoscopy is an endoscopic technique for direct visualization in the diagnostic workup of PCLs and can be impactful when performed intraoperatively.^1^ It is helpful in the investigation of MD-IPMNs and distinguishing them from benign causes of MPD dilation such as chronic pancreatitis. It carries a diagnostic accuracy of 88% in differentiating MD-IPMNs and identifying malignancy. The presence of fish-egg-like protrusion on vascular images, villous protrusions, or vegetative protrusions was 78% specific and 68% sensitive for malignancy.^32^ Using narrow band imaging improves diagnosis by providing better visualization of surface structure and vascular patterns.^33^ Biopsies can also be obtained at the time. Targeted tissue and fluid are procured to sample neoplastic cells and rule out dysplasia.^30^ The fluid can easily be sent off for molecular marker analysis for risk stratification.

Preoperative pancreatoscopy has been shown to change surgical management. main duct IPMNs are either diffuse or segmental and may consist of skip lesions not well seen on cross-sectional imaging. In a pilot study of 46 patients who underwent pancreatoscopy, 65.2% had a change in surgical strategy with conversion to extended resection or segmental resection. However, pancreatoscopy is associated with a post-ERCP pancreatitis risk of 2.5%, plus other adverse effects, including duodenal perforation and bleeding.^1^

## EUS-Guided Cyst Ablation

A minimally invasive approach may be employed for PCLs that demonstrate local growth but are considered unresectable. One such method is EUS-guided cyst ablation, which entails draining the cyst and injecting either ethanol or chemotherapeutic drugs such as paclitaxel and gemcitabine into lesions, albeit with modest efficacy.^30^ Like nCLE, it is experimental. Patients may opt to pursue this treatment option if they are reluctant or unable to have surgery. This treatment approach is relatively uncommon and does not constitute definitive management. However, it permits the preservation of exocrine and endocrine parenchymal tissue. Suspected mucinous PCLs between 2 and 6 cm with cytology negative for malignancy are eligible for cyst ablation. Short-term follow-up appears encouraging.^34^ Still, durability and long-term effects remain undescribed. Therefore, randomized controlled trials, long-term studies, and guidelines for follow-up are still needed. The biggest challenges resulting from cyst ablation include how and when to surveil post-ablative patients.^30^ The natural history of treated lesions is currently indeterminate, and questions remain about what deems an effective ablation. For example, the development of fibrosis at the site of a presumed successful ablation can obfuscate future assessments. Furthermore, PCLs or adenocarcinoma can continue to appear in untreated areas of the pancreas, which exposes another limitation of ablation. This concept is known as a field defect and bears relevance for patients after procedures such as partial pancreatectomy. According to guidelines from Europe, the American College of Gastroenterology, and the IAP, there is too little evidence for cyst ablation’s routine use.^2,3,11,12^

## Surgery

Surgical resection is recommended for malignant or premalignant PCLs with concern for HRS or malignant transformation.^2,11,12,35^ All guidelines also recommend reserving surgical resection for experienced surgeons at high-volume centers after review by a multidisciplinary team with pancreatic expertise as long as the patient is surgically fit.^26^ This is due to the growing evidence that the mortality rates at low-volume centers are significantly higher than at high-volume centers. In younger patients who would otherwise need lengthy surveillance, surgical resection is also favored, but decisions are individualized.^2,3^ Surgery for an SCA is indicated for cysts that become symptomatic or grow considerably with time.^14^ Choosing surgical treatment is seldom clear-cut but is contingent upon the extent and anatomic location of a lesion. Table 3 summarizes the suggested indications for surgical consultation for PCLs, which are predicated on the guidance of multiple organizations.

There are different approaches to surgical procedures. Resections can be performed via a standard open approach. However, minimally invasive approaches have become more popular with advances in technology and have yielded better outcomes. Furthermore, the type of surgical resection is dependent on the location and extent of PCL. Pancreatic cystic lesions located within the head, neck, or uncinate process of the pancreas require pancreaticoduodenectomy. This entails resecting the pancreatic head, duodenum, part of the jejunum, the common bile duct, gallbladder, part of the stomach, and lymph nodes. Mortality has been reported to be 1%-5%^36,37^ while morbidity is up to 60%.^38^

Recent trends indicate that parenchyma-sparing resections and minimally invasive procedures are performed more frequently to cut down rates of post-operative pancreatic insufficiency and reduce the surgical impact of such operations.^39^ Distal pancreatectomy is reserved for PCLs in the body or tail of the pancreas. The body and tail of the pancreas are removed and, in most cases, the spleen as well. However, spleen-preserving distal pancreatectomy may be considered when malignancy is not suspected. While it is associated with a lower mortality of <1% compared to pancreaticoduodenectomy, it still has a morbidity of about 30%.^40^ Patients who have undergone distal pancreatectomy may be fortunate enough to avoid severe pancreatic insufficiency given parenchymal preservation. This is because the vast majority of the pancreas is composed of exocrine tissue, while only a minute percentage derives from endocrine structures. Even so, 20% of patients without other pancreatic conditions will develop new-onset DM after partial pancreatectomy for PCLs within 2 years.^41^

Central pancreatectomy removes the neck and proximal body of the pancreas and preserves the head and tail. This is less extensive than surgical resection, reserved for PCLs located within the neck of the pancreas, and therefore allows improved long-term function of the pancreas. However, it has been associated with a mortality of 0.5% and morbidity of 51%^42^ and is therefore performed selectively.

Alternatively, total pancreatectomy is the removal of the entire pancreas. This operation carries a mortality rate of 14%^43^ and, in cases of IPMNs, is commonly associated with the likelihood of positive resection margins.^39^ While most surgeons avoid this procedure, it is sometimes warranted in cases of diffuse disease, especially with diffuse MD-IPMNs. Total pancreatectomy risks iatrogenic exocrine pancreatic insufficiency, a syndrome of maldigestion.^26^ It also risks type 3C diabetes, otherwise known as pancreatogenic diabetes, with eventual insulin dependence. Therefore, age and comorbidities should be considered when deciding whether to perform this procedure.

Enucleation is the selective resection of a pancreatic lesion and is associated with improved operative outcomes.^44^ It allows the preservation of pancreatic tissue and function. However, this is only indicated for small, benign PCLs that do not involve the MPD or side branches and is more commonly performed for small PNETs.

Recent data affirms that surgical resection can be performed with a diagnostic accuracy of 80%, minimal mortality, and tolerable morbidity. The concordance of preoperative and final histopathologic diagnoses has risen in the last three decades from 45% to 80%.^14^ Overall, pancreatic resection has several complications, including delayed gastric emptying, abscess, postoperative bleeding, and postoperative pancreatic fistula formation. Postoperative pancreatic fistulas are more likely to form after surgery for PCLs than for pancreatic adenocarcinoma and are more common with central pancreatectomies.^45^ Grade 3 pancreatic fistulas can cause significant morbidity, leading to additional procedures and lengthened hospital stays. Long-term complications also include endocrine and exocrine insufficiency.

Distal pancreatectomy also risks diagnostic inaccuracy as high as 30%, which could be partly attributable to the inclination of surgeons to resect distal pancreatic lesions without preoperative certainty about cyst characterization. In this study, “delayable surgery” was classified as benign histology without an absolute indication, and the overall risk of unnecessary immediate surgery at a high-volume referral center was 22%.^14^ About 10% of patients in another study had surgery for benign lesions that were believed to be of malignant potential or cancerous.^46^ After surgical resection, the 5-years survival of patients with non-malignant IPMNs has been reported as 94%-100%, while patients with non-malignant MCNs have a 97% 5-years survival rate.^45^ Given pancreatic surgery’s morbidity and mortality, the decision to resect asymptomatic PCLs should be based on the operative mortality and the probability that the lesion is malignant and its resection will prolong survival.

## Future Prospects

Among the emergent uses of artificial intelligence (AI) are AI algorithms for risk stratification of PCLs. Multiple teams have developed systems to classify or diagnose PCLs with the hope of increasing diagnostic accuracy.^47^ Several studies have already shown that AI-powered quantitative analysis of PCL epithelium during EUS-nCLE outperformed the current standard of care in diagnosing HGD/adenocarcinoma and low-grade dysplasia in IPMNs.^48-50^ However, a great deal of image pre-processing is still generally involved before AI is able to interpret information. Therefore, the most apparent limitations of AI are currently the inevitable labor and human element behind priming such systems. The ultimate goal is to shift toward more independent and predictive algorithms that eliminate or reduce the specialist’s workload.^47^

## Conclusion

Pancreatic cystic lesions are most commonly detected as incidentalomas. They can be stratified on a spectrum of malignant potential based on their imaging and clinicopathologic characteristics. It is well established among existing guidelines that certain features of PCLs confer a higher risk of malignancy in the future, chiefly dilation of the MPD, intramural solid/mass component, cyst size >3 cm, and abnormal or suspicious cytology findings. The diagnosis of PCLs improves significantly with the addition of cyst fluid and molecular marker analysis, as imaging alone is not enough to confidently diagnose such lesions. The current guidelines are limited in that they are sometimes at odds with each other and based on expert opinion and low-quality evidence. There may be benefits to streamlining and eventually integrating global guidelines in the future. More high-quality research is needed on various aspects of PCLs for the evolution of clinical practice, which is increasingly precision-oriented and will likely enlist AI.

## Figures and Tables

**Table 1.** Pancreatic Cystic Lesion (PCL) Morphologies

			Endoscopic Features		
PCL Type	Common Location	MPD Communication	Endosonographic	Endomicroscopic	Cyst Fluid Analysis
Pseudocyst	Variable	Possible	Thick-walled and anechoic. May contain debris. Septa are infrequent	Lacking blood vessels. Dark background speckled with bright white	Cytology: inflammatory cells (neutrophils, macrophages, histiocytes). Fluid markers: low CEA (<0.5), high amylase
SCA	Body, tail	None	Microcystic with central scar. May be oligocystic or, less commonly, macrocystic. Possible solid portion. Solitary unless diagnosis of VHL present	Superficial vascular network, i.e., fern-pattern vascularity	Cytology: cuboidal epithelium with abundant cytoplasm. Fluid markers: Low CEA (<0.5), VHL mutation
IPMN	Variable	Definite	Multifocal. Unilocular versus septated. Fish mouth papilla pathognomonic for main duct IPMN	Horizontally-oriented epithelial bands with papilla. Central fibrovascular core	Cytology: columnar cells, mucin (high viscosity). Fluid markers: high CEA, KRAS/GNAS mutation
MCN	Body, tail	None	Solitary. Unilocular versus septated. Peripheral calcification in 10%-25%	Horizontally-oriented epithelial bands without papilla	Cytology: columnar cells, mucin (high viscosity). Fluid markers: high CEA, *KRAS* mutation
PanNET	Variable	None	Solid and cystic mass. Unilocular or multilocular. Solitary unless diagnosis of MEN is present	Trabeculated with organized clusters of cells surrounded by stroma	Cytology: homogeneous cells, round nuclei, staining for chromogranin and synaptophysin
SPN	Body, tail	None	Solid, mixed solid or cystic mass. Peripheral or central calcification (acoustic shadowing)	Trabeculated with organized clusters of cells surrounded by stroma	Cytology: monomorphic cells with pseudopapillary structures. Fluid markers: CTNNB1 mutation

CEA, carcinoembryonic antigen; CTNNB1, Catenin beta-1; GNAS, guanine nucleotide-binding protein (G protein), alpha-stimulating activity polypeptide 1; IPMN, intraductal papillary mucinous neoplasm; KRAS, Ki-ras2 Kirsten rat sarcoma viral oncogene homolog; MCN, mucinous cystic neoplasm; MEN, multiple endocrine neoplasia; MPD, main pancreatic duct; PanNET, pancreatic neuroendocrine tumor; SCA, serous cystadenoma; SPN, solid pseudopapillary neoplasm; VHL, Von Hippel-Lindau syndrome.

**Table 2.** Pooled Specificities and Sensitivities for Distinguishing Mucinous from Non-Mucinous Cysts

	Approximate Cut-Off	Pooled Specificity (%)	Pooled Sensitivity (%)
Amylase^17^	<250 U/L	98	44
Carcinoembryonic antigen^18^	>192 ng/mL	87	58
Intracystic glucose^18^	<50 ng/dL	93	76

**Table 3.** Summary of Indications for Surgical Consultation from Major Gastroenterological Organizations

Guiding Organization	Features that Trigger Surgical Evaluation		
	Clinical	Laboratory	Imaging
ACG	New-onset DM, obstructive jaundice from cyst, acute pancreatitis from cyst	Elevated serum CA 19-9 level, cytology: HGD or invasive disease	PCL growth exceeding 3 mm/year; dilation of MPD concerning for cyst growth by >3 mm/year, solid component, MPD dilation exceeding 5 mm, focal dilation of MPD concerning for MD-IPMN or obstructing lesion; IPMN or MCN size >3 cm
ACR	Jaundice resultant from cyst	None	Immediate surgical consultation if enhancing mural nodules or MPD ≥ 10 mm. Consider surgery for: mural nodule, MPD ≥ 7 mm, thickened/enhancing cyst wall, or symptomatic SCA ≥ 4 mm
AGA	None	Cytology: HGD or invasive malignancy	Dilated MPD, PCL size >3 cm
**European**			
Absolute indications for surgery	Jaundice resultant from cyst	Cytology: HGD or invasive disease	Solid mass, enhancing mural nodule >5 mm, MPD dilation exceeding 10 mm
Relative indications for surgery	New-onset DM, acute pancreatitis induced by IPMN	Serum CA 19-9 > 37 U/mL	Growth rate >5 mm per year, MPD dilation between 5 and 9 mm, PCL diameter exceeding 4 cm; enhancing mural nodule <5 mm
**IAP/Kyoto (2023) **	Jaundice associated with lesion in the head of pancreas. Acute pancreatitis^, new-onset DM^ (consideration, but not definite)	Cytology: HGD or invasive disease. Elevated CA 19-9^ (consideration, but not definite)	Enhancing mural nodule >5 mm or solid component, MPD > 10 mm. Presence of multiple^: enhancing mural nodule of ≥5 mm, thickened or enhancing cyst walls, MPD 5-9 mm, abrupt change in caliber of MPD with distal pancreatic atrophy; cyst growth rate ≥2.5 mm/year

^ Worrisome feature.

ACG, American College of Gastroenterology; ACR, American College of Radiology; AP, acute pancreatitis; ACR, American College of Radiology; AGA, American Gastrointestinal Association; AP, acute pancreatitis; BD-IPMN, branch-duct intraductal papillary mucinous neoplasm; CA, cancer antigen; DM, diabetes mellitus; HGD, high-grade dysplasia; HRS;HRS, high-risk stigmata; IAP, International Association of Pancreatology; MCN, mucinous cystic neoplasm; MD-IPMN, main duct intraductal papillary mucinous neoplasm; MPD, main pancreatic duct; PCL, pancreatic cystic lesion.

## Data Availability

The data that support the findings of this study are openly available.
